# 

**Published:** 1876-11

**Authors:** 


### BOOKS AND PAMPHLETS RECEIVED.

### BOOKS.

A Practical Treatise on the Diseases, Injuries and Malformations of the Urinary Bladder, Prostate Gland and the Uretha. By S. D. Gross, M.D., etc. Third edition, revised and edited by S. W. Gross, M.D., etc.

Chemistry: General, Medicinal and Pharmaceutical, including the Chemistry of the U. S. Pharmacopœia, etc. By John Attfield, Ph. D., F.C.S., etc.

Compend of Histology: Twenty-four Lectures. By Heinrich Frey, Professor. Translated by Geo. R. Cutler, M.D., etc. Illustrated.

PAMPHLETS.

Transactions of the Medical Society of New Jersey, 1876.

The modern methods of Examining the upper air passages. By G. M. Lefferts, M.D., etc., being No. VI. of Vol. II., of "a series of American Clinical Lectures." Edited by E. C. Seguin, M.D., etc.

### ANNOUNCEMENTS FOR THE MONTH.

**MONDAYS.** SOCIETIES.

*Mondays, Nov.* 6 *and* 20.—Chicago Medical Society. Regular meetings.
*Mondays, Nov.* 13 *and* 27.—Chicago Soc. of Phys. and Surgeons. Regular meetings.

CLINICS. *Every Monday.*

At Eye and Ear Infirmary, 2 P. M.—Prof. Holmes.
At Central Dispensary (Wood and Harrison sts.), 2 P.M. *Gynecological*—Dr. Adolphus.
At Mercy Hospital—2 P. M., *Surgical*, Prof. Andrews.
At Rush College—2¼ P. M., *Medical*, Dr. Bridge.
At Chicago College, 2 P. M *Gynecological*—Prof. Merriman.

LECTURES. *Every Monday.*

At Rush Medical College (Harrison and Wood sts.)—9½ to 12½ o'clock, Profs. Freer, Lyman and Powell; 4 to 6, Profs. Gunn and Haines. At Chicago College—8½ to 12½, Profs. Jewell, Hyde, Hatfield and Bond; 3 to 6, Profs. Nelson and Davis, Quine and Andrews, and Roler. At Woman's College—8½ to 11½, Profs. Hotz, Marguerat and Earle; 3 to 5, Profs. Paoli and Stevenson.

**TUESDAYS.** SOCIETIES.

*Tuesday, Nov.* 14.—Academy of Science. Regular meeting at 8 P. M. (263 Wabash av.)

CLINICS. *Every Tuesday.*

At County Hospital—2 P. M., *Medical*, Prof. Ross. 3 P. M., *Surgical*, Prof. Powell.
At Mercy Hospital—2 P. M., *Medical*, Prof. Hollister.
At Chicago College—2 P. M., *Gynecological*, Prof. Roler.
At Central Dispensary—2, *Surgical*, Dr. Graham. 3, *Diseases of Chest*, Dr. Ingals.

LECTURES. *Every Tuesday.*

At Rush College—9½ to 12½, Profs. Miller, Allen and Parkes; 4 to 6, Profs. Gunn and Haines. At Chicago College—8½ to 12½, Profs. Jewell, Quine, Merriman and Bond; 3 to 6, Profs. Johnson and Nelson, Andrews and Hatfield, and Byford. At Woman's College—9 to 12, Profs. Bartlett, Bogue and Fitch; 3 and 5, Profs. Thompson and Macdonald.

**WEDNESDAYS.** CLINICS. *Every Wednesday.*

At County Hospital—2 P. M., *Gynecological*, Prof. Fitch; 2 P. M., *Ophthalmological*, Dr. Montgomery.
At Chicago College—2 P. M., *Gynecological*, Prof. Nelson.
At Mercy Hospital—2 P. M., *Ophthalmological*, Prof. Jones.
At Central Dispensary—2 P. M., *Gynecological*, Prof. Etheridge; 3 P. M., *Dermatological*, Dr. Maynard.

LECTURES. *Every Wednesday.*

At Rush College—9½ to 12½, Profs. Freer, Allen and Parkes; 4 to 6, Profs. Etheridge and Haines. At Chicago College—8½ to 12½, Profs. Hyde, Isham, Hatfield and Bond; 3 to 6, Profs. Davis and Curtis, Quine and Jones, and Roler. At Woman's College—9 to 12, Profs. Marguerat, Curtis and Blake; 3 to 6, Paoli, Graham and Stevenson.

**THURSDAYS.** CLINICS. *Every Thursday.*

At Mercy Hospital—2 P. M., *Medical*, Prof. Davis.
At Rush College—2 P. M., *Medical*, Prof. Ross; 3 P. M., *Diseases of the Nervous System*, Prof. Lyman.
At Chicago College—2 P. M., *Gynecological*, Prof. Merriman.
At Central Dispensary—2 P.M., *Surgical*, Dr. Graham; 3, *Diseases of Chest*, Dr. Ingals.

LECTURES. *Every Thursday.*

At Rush College—9½ to 12½, Profs. Miller, Allen and Parkes; 4 to 6, Profs. Gunn and Etheridge. At Chicago College—8½ to 12½, Profs. Hollister, Isham, Merriman and Bond; 3 to 6, Profs. Johnson and Nelson, Andrews and Hatfield, and Byford. At Woman's College—9 to 12, Profs. Dyas, Bogue and Fitch; 5, Prof. Macdonald.

**FRIDAYS.** SOCIETIES.

*Friday, Nov.* 10.—State Microscopical Society of Illinois. Regular meeting, 8 P. M.

CLINICS. *Every Friday.*

At County Hospital—2 P. M., *Medical*, Prof. Ross; 3 P. M., *Surgical*, Prof. Powell.
At Mercy Hospital—2 P. M., *Medical*, Prof. Davis.
At Chicago College—2 P. M., *Gynecological*, Prof. Roler.
At Central Dispensary—2 P. M., *Gynecological*, Dr. Adolphus.

LECTURES. *Every Friday.*

At Rush College—9½ to 12½, Profs. Freer, Allen and Parkes; 4 to 6, Profs. Gunn and Holmes. At Chicago College—8½ to 12½, Profs. Hollister, Isham, Hatfield and Bond; 3 to 6. Profs. Davis and Curtis, Jones and Quine, and Roler. At Woman's College—9 to 12, Profs. Bartlett, Marguerat and Curtis; 5, Prof. Stevenson.

**SATURDAYS.** CLINICS. *Every Saturday.*

At Chicago College—2 P. M., *Surgical*, Prof. Andrews or Isham; *Gynecological*, Prof. Nelson; 3 P. M., *Medical*, Prof. Johnson.
At Rush College—2 P. M., *Surgical*, Prof. Gunn.

LECTURES. *Every Saturday.*

At Rush College—9½ to 12½, Profs. Miller, Lyman and Etheridge. At Chicago College 8½ to 11½, Profs. Hollister, Quine and Hyde; 3 to 6, Profs. Nelson and Johnson, Andrews and Quine and Byford. At Woman's College—9 to 11, Profs. Earle and Fitch; 3 and 5, Profs. Paoli and Macdonald.

At all the above named Clinics visiting regular practitioners are, we believe, admitted.
At the South Side Dispensary (Chicago College) there are six daily special Clinics, for sections of the classes of the Chicago College.

